# Social disappointment and partner presence affect long-tailed macaque refusal behaviour in an ‘inequity aversion’ experiment

**DOI:** 10.1098/rsos.221225

**Published:** 2023-03-01

**Authors:** Rowan Titchener, Constance Thiriau, Timo Hüser, Hansjörg Scherberger, Julia Fischer, Stefanie Keupp

**Affiliations:** ^1^ Cognitive Ethology Laboratory, Deutsches Primatenzentrum GmbH, Kellnerweg 4, 37073 Goettingen, Germany; ^2^ Neurobiology Laboratory, Deutsches Primatenzentrum GmbH, Kellnerweg 4, 37073 Goettingen, Germany; ^3^ Institute of Psychology, University of Goettingen, Waldweg 26, 37073 Goettingen, Germany; ^4^ Leibniz ScienceCampus Primate Cognition, 37077 Goettingen, Germany; ^5^ Université Paris Nord, 99 Avenue Jean Baptiste Clément, 93430 Villetaneuse, France; ^6^ Faculty of Biology and Psychology, University of Goettingen, 37077 Goettingen, Germany; ^7^ Department for Primate Cognition, University of Goettingen, 37077 Goettingen, Germany

**Keywords:** social disappointment, inequity aversion, long-tailed macaques, *Macaca fascicularis*

## Abstract

Protest in response to unequal reward distribution is thought to have played a central role in the evolution of human cooperation. Some animals refuse food and become demotivated when rewarded more poorly than a conspecific, and this has been taken as evidence that non-human animals, like humans, protest in the face of inequity. An alternative explanation—social disappointment—shifts the cause of this discontent away from the unequal reward, to the human experimenter who could—but elects not to—treat the subject well. This study investigates whether social disappointment could explain frustration behaviour in long-tailed macaques, *Macaca fascicularis*. We tested 12 monkeys in a novel ‘inequity aversion’ paradigm. Subjects had to pull a lever and were rewarded with low-value food; in half of the trials, a partner worked alongside the subjects receiving high-value food. Rewards were distributed either by a human or a machine. In line with the social disappointment hypothesis, monkeys rewarded by the human refused food more often than monkeys rewarded by the machine. Our study extends previous findings in chimpanzees and suggests that social disappointment plus social facilitation or food competition effects drive food refusal patterns.

## 1. Introduction

Humans attend keenly to resource division [1]. We routinely compare our own pay-offs with those of the people around us, and from a young age, we show a strong preference for fair resource division [2]. A number of principles govern how we divide resources [3]. In some contexts, we value equality, in others we accept merit- or need-based resource distribution (e.g. [4]). When we consider resources to have been unfairly or mis-allocated, we make this known—we protest. Such policing behaviour is thought to have contributed to the emergence of cooperation in humans [5–8].

Motivated by the fact that non-human species—like humans—profit from cooperation (e.g. [9,10]), scientists have set out to investigate whether animals also protest when faced with disadvantageous food distribution. A widely used way to probe how animals respond to unequal food distribution is the token exchange paradigm developed by Brosnan & de Waal [11]. In this experimental set-up, two conspecifics—a subject and a partner—alternately interact with a human experimenter. The monkeys take turns to hand a token to the experimenter and receive a food reward. In an inequality condition, subjects receive low-value (LV) food for their work, while observing that the partner receives high-value (HV) food. In a control equality condition, the two monkeys both receive the same quality of food. Brosnan and de Waal reported that in capuchin monkeys, subjects rejected food rewards more often in the inequality as opposed to the equality condition. This pattern of behaviour has now been demonstrated in a number of primate [12–14] and non-primate species [15,16]. While the pattern of refusal behaviour appears robust (but see [17,18]), its interpretation is less clear.

The initial interpretation was that the animals' refusal behaviour was due to social comparison with the partner monkey, challenging the idea that humans’ sensitivity to inequitable reward distributions is unique ([11], theoretical considerations refined in [6]). This interpretation of the results has been termed the ‘inequity aversion’ hypothesis. The inequity aversion hypothesis contends that subjects track and compare their reward scheme with that of the partner. Subjects have expectations about which food they should receive after observing a conspecific receiving HV rewards for the same work, and, as a form of protest, they refuse LV rewards. In line with the inequity aversion hypothesis, a number of species that do not share resources such as territory, food, or support during conflict (e.g. orangutans (*Pongo pygmaeus*), squirrel monkeys (*Sairmiri* spp.) and cleaner fish (*Labroides dimidiatus*), also do not show refusal behaviour akin to that shown by capuchin monkeys [19–21]. Brosnan & de Waal's [11] conclusion has nonetheless proven contentious [22]. For instance, whether capuchin's refusal behaviour is appropriately likened to that of humans has been questioned [22] although tests have since shown that LV rewards are indeed refused by adult humans in such a scenario [23].

Since the publication of the original study [11], a number of alternative hypotheses have been put forward to explain subjects' refusal behaviour in the token exchange paradigm. Two alternative explanations revolve around food: the food expectation hypothesis and the frustration hypothesis. The food expectation hypothesis contends that the mere sight of HV food in the inequality condition is sufficient to elicit refusal behaviour [24]. The sight and proximity of a coveted reward lead monkeys to form expectations about receiving these rewards. Support for the food expectation hypothesis has been mixed; the hypothesis has had its proponents (e.g. [25,26]) but also its challengers (e.g. [27,28]). Bräuer *et al*. [25] reported that contrary to the predictions of the inequity aversion hypothesis, apes refuse food *less* often in the presence of a well-rewarded conspecific compared with when alone, and in addition to this, they emphasize the fact that their chimpanzee subjects (they tested four ape species) showed overt begging behaviour. As this begging behaviour was directed toward the human, Bräuer and colleagues interpreted this behaviour display as evidence in support of the food expectation account. Dubreuil and colleagues tested capuchin monkeys in four experimental conditions to investigate the potential effects that the handling and sight of HV food may have on refusal patterns of LV food [26]. They reported that food refusals were at their peak when HV food was visible and accumulating, as opposed to when it was briefly handled and then hidden or consumed by a partner monkey. Challenging the growing number of studies endorsing the food expectation hypotheis, van Wolkenten and colleagues re-ran the original Brosnan and de Waal [11] experiment with a larger sample size and rigorous control conditions, again finding in favour of the inequity aversion account [28]. Brosnan and colleagues have also defended their inequity aversion interpretation, criticizing the methods used to gather evidence in support of the food expectation hypothesis—for instance, the fact that subjects were *gifted*, rather than made to work for rewards; the requirement that a subject expends effort to acquire the reward is considered crucial to the inequity aversion hypothesis [27].

As touched upon earlier, the food expectation hypothesis is but one of two hypotheses to revolve around food concerns. A second hypothesis, the frustration hypothesis, contends that a subject's past experience in receiving a preferred reward for a task creates an expectation to receive this reward [29]. Roma and colleagues highlighted a potential confound in Brosnan and de Waal's [11] study, namely that in order to increase their sample size, the same monkeys were used for the subject and partner roles [29]. To explore a potential effect of frustration on refusal behaviour, Roma and colleagues tested eight capuchins in a conceptual replication and reported that when the subject–partner role-switches were eliminated from the experimental design, their food refusal rates in the inequity conditions were low compared with the food refusal rates reported by Brosnan and de Waal. As with the food expectation hypothesis, the frustration hypothesis has been the subject of considerable debate (e.g. [30]; [28]). Brosnan and de Waal re-analysed a subset of their 2003 data with the frustration hypothesis in mind and found no evidence to indicate that refusal rates were affected by previous receipt of HV rewards [30]. In their 2007 paper, van Wolkenten and colleagues also checked for evidence that might support the frustration hypothesis and reported that subjects' refusal rates did not fluctuate as a function of the value of the food item the subject had received in a prior exchange [28].

Recently, Engelmann and colleagues put forward a third explanation—the social disappointment hypothesis [31]. This hypothesis highlights the role of the human experimenter. It shifts the cause of frustration away from the food and the well-rewarded partner, to the deliverer of the food. The social disappointment hypothesis contends that subjects compare how they are treated, with how they could be treated. By refusing to take the reward in the unequal condition, the subject is expressing its dissatisfaction with the human who gives LV instead of HV rewards.

Engelmann and colleagues investigated the social disappointment hypothesis in nine chimpanzees using a tool-exchange task. Subjects inserted a tool into an apparatus in order to obtain a food reward. Either a human or a machine—technically, an out-of-view experimenter operated the apparatus by pulling strings—rewarded the subject for a successful exchange. Subjects worked either alone or next to a partner alternately engaged in the same task. Chimpanzees that interacted with the human rather than the machine refused food more frequently. A secondary finding was that partner presence resulted in fewer rather than more food refusals compared with the no-partner condition, a converse pattern to that expected under the inequity aversion hypothesis. This experiment was the first study to systematically manipulate the nature of the distributor in a token exchange paradigm and provide clear support for the social disappointment explanation.

At present, the social disappointment hypothesis has only been tested in chimpanzees. Motivated to provide a broader comparative perspective, our study aimed to extend those findings. We tested the social disappointment hypothesis in a catarrhine monkey, the long-tailed macaque, *Macaca fascicularis*. Long-tailed macaques show a number of cooperative behaviours (see [14]), for example they form defensive coalitions [32] and they reciprocate beneficial grooming behaviour [33]. This species has been tested in an inequity aversion paradigm, and subjects refused food more often under inequality as opposed to equality conditions under conditions of moderate effort [14]. Motivated to establish whether Engelmann and colleagues’ hypothesis would extend to a second species that is thought to show inequity-averse protest behaviour, we set out to investigate whether long-tailed macaque refusal behaviour could be explained by social disappointment.

Our study design is conceptually faithful to Engelmann *et al*.'s study, including details of the experimental design. However, as our monkeys are not great tool users, we had to devise a different experimental set-up. Subjects had to pull a lever and in exchange they received a food reward. Either a human or a machine rewarded the subject for pulling the lever (implemented as a between-subject factor) and the subject either worked alone or next to a partner engaged in the same task (within-subject factor). In contrast with the chimpanzee study, our subjects additionally experienced a control condition in which the partner monkey received the same LV food as the subject.

This study allowed us to contrast the different predictions derived from the theoretical considerations outlined above. If inequity aversion motivates refusal behaviour, the distributor should be irrelevant and monkeys should refuse food most often in the test conditions where the well-rewarded conspecific is present. If food expectation motivates refusal behaviour, then monkeys should refuse LV food equally often in all test conditions. Finally, if social disappointment explains refusal behaviour, monkeys assigned the human distributor should refuse food more often than those assigned the machine distributor irrespective of partner presence and treatment. A control condition was included to distinguish between these different accounts using additional evidence. The inequity aversion hypothesis predicts no refusals when both individuals receive the same LV food, irrespective of distributor. The food expectation hypothesis predicts refusal in all conditions where HV food is visible, which is the case for both control conditions. The social disappointment hypothesis predicts more refusals in the human distributor control condition compared with the machine control condition.

## 2. Material and methods

### 2.1. Subjects

Fourteen monkeys took part in the study, 12 as subjects (1–7 years of age, median age = 3.4 years, 7 females) and two in the role of partner monkey (both 6 years of age, both male). To simplify the statistical analysis, we originally intended to use only one partner monkey (Ilja). Ilja was selected as he was mid-ranking (assessed informally in reports from several caretakers and researchers) and unrelated to any subjects. Unfortunately Ilja was removed from the social group part-way during the experiment. In an attempt to minimize a potential effect of partner identity, we took care to select a second partner monkey (Linus) for his similarity to Ilja; Linus was also mid-ranking and unrelated to the subjects. The monkeys were housed in a social group of 37 individuals at the German Primate Center in Göttingen. All 12 test subjects completed the test conditions and 10 of the original 12 subjects also completed the control condition. Participation in the study was voluntary and dependent young often remained with their mothers during testing. For detailed information about subjects and condition assignment, refer to electronic supplementary material, table S1.

### 2.2. Materials

A table apparatus was attached to the subject's test compartment (electronic supplementary material, figure S1). A red light was fixed to the table, and there was a lever within reach of the monkey. The red light served as a cue for the monkey; when the light was turned off, the lever was blocked, and when the red light was turned on, the monkey was able to pull the lever. Two red carousels were positioned on a plexiglass platform in front of and equidistant to the red light. On top of each carousel was a transparent cylinder that was used to display food. Each carousel had small compartments that could hold food. An identical apparatus was attached to the neighbouring test compartment, which either held a partner monkey or was empty (as per condition requirements). In test conditions, the subject's apparatus was not accessible to the partner monkey and vice versa. The subject had an unobstructed view of the partner's table.

### 2.3. Design

**Figure 1.**
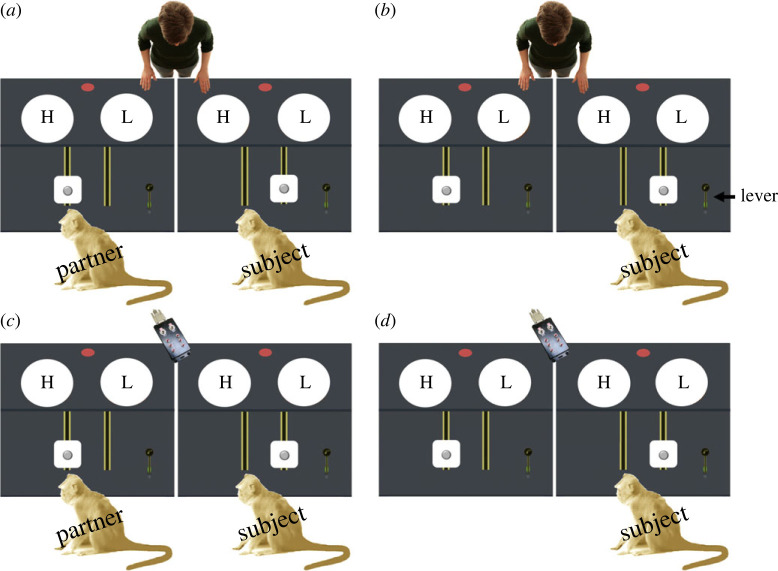
The experimental design: (*a–d*) depict test conditions. (*a*) Human distributor/partner present, (*b*) human distributor/partner absent, (*c*) machine distributor/partner present and (*d*) machine distributor/partner absent. Circles containing ‘H’ represent carousels containing HV food and circles containing ‘L’, represent carousels containing LV food.

We implemented the same 2 × 2 factorial design used in the [31] study (figure 1). *Distributor* (human/machine) was a between-subject factor and *partner presence* a within-subject factor. In addition to the original conditions, we ran a control condition where both monkeys received LV food. The subjects proceeded through five stages during the course of the study: (1) a food preference test, (2) lever-training, (3) a four-step familiarization procedure, (4) the original test conditions and (5) the additional control condition. Information about steps (1) to (3) can be found in §4 of the electronic supplementary material. Stages (4) and (5) are described below.

## 3. Methods

### 3.1. Test conditions

At the start of the familiarization procedure, subjects were assigned a distributor (human or machine) and they remained with this distributor for the duration of the study. Seven subjects were assigned the human distributor (three males) and five subjects the machine distributor (two males; for details see electronic supplementary material, table S1). Each subject experienced two types of test conditions, a *partner present* and a *partner absent* condition (ABAB presentation, order counterbalanced and in total four sessions per condition). Subjects had a maximum of one session per day, with 12 trials comprising a session (see electronic supplementary material, table S2 for exceptions due to technical failures or drop-outs). Note that in each test condition, both HV and LV food were on display and the food item offered to the monkey became clear only after s/he had pulled the lever. The subject and partner interacted alternately with their respective tables.

In the human distributor condition, there was one person in the test room. After the subject pulled the lever, the LV food carousel rotated. The human reached into the food container sitting on the carousel and took out a food item, which she placed onto a small platform that travelled on a conveyor belt toward the subject. By contrast, in the machine condition, the subject (subject and partner, in the *partner present* condition) was (were) alone in the test room. After the subject had pulled the lever, the LV food carousel rotated. Rotation of the carousel caused a food item to drop through a hole and fall onto the small platform, which then travelled on a conveyor belt toward the subject. Although there were common elements to the food delivery (rotating carousel, platform conveys the food forwards), the key element of difference between the two conditions was the presence/absence of the human and the fact that in the human distributor condition, food delivery depended on the actions of an experimenter. This is in keeping with Engelmann *et al*.'s study in which the food delivery process was kept constant, while the presence/absence of a human was manipulated.

***Human distributor/partner present* condition** (figure 1*a*; for video exemplar see electronic supplementary material, video S1). Prior to each *human distributor/partner present* condition, E1 filled one transparent cylinder on each table with LV food and one with HV food (side counterbalanced between sessions). E1 left the room and E2 entered to stand behind the partner's table. E1 monitored the activity in the test room via video and switched the light on the partner's table to on via remote control. If the partner pulled the lever within 120 s after the light went on, the cylinder containing the HV food rotated and the platform on the conveyor belt moved towards the partner. E2 took an item of HV food from the cylinder and placed it on the moving platform. Once the platform reached the end of the track, the monkey had 2 s to take the food before the platform travelled back to the start position. If the monkey refused to take the food, it was mechanically swept off the platform into a bag under the table. Once the platform had returned to the start position, E2 moved to stand behind the subject's table and the same procedure was carried out for the subject with the exception that this monkey was only ever offered LV food. If the lever was not pulled within 120 s, the light on the table went out and E2 moved to service the other table. E2 alternated between the two tables until each monkey had completed 12 trials.

***Human distributor/partner absent* condition** (figure 1*b*; electronic supplementary material, video S2). No partner was present in this condition. This aside, the sequence of events was identical to those of the *human distributor/partner present* condition. Given that no monkey was present to take the HV food in the partner's compartment, the partner's platform returned to the start position laden with food which was mechanically swept into the bag under the table. E2 alternated between the partner and the subject table until the subject had completed 12 trials.

***Machine distributor/partner present* condition** (figure 1*c*; electronic supplementary material, video S3). Prior to each session, E1 filled each transparent cylinder on the tables with food (one LV- and one HV-cylinder per table) and then baited 12 carousel compartments with the respective food (presentation side counterbalanced). E1 then left and both E1 and E2 remained outside the room for the duration of the session. The sequence of events which followed was identical to the *human distributor/partner present* condition except that now the machine was responsible for loading the platform that travelled within reach of the monkey. In other words, the key difference between the two distributor conditions was the presence/absence of E2 in the test room, and the fact that food delivery depended on E2 in the human distributor condition. E1 alternated via remote control which table was active (indicated by the lamp), until each monkey had completed 12 trials.

***Machine distributor/partner absent* condition** (figure 1*d*; electronic supplementary material, video S4). No partner was present in this condition. This fact aside, the sequence of events in this condition was identical to that in the *machine distributor/partner present* condition. Food on the partner's platform returned to the start position and was mechanically swept into the bag under the table. E1 alternated via remote control which table was active until the subject had completed 12 trials.

### 3.2. Control condition

Following the test conditions, 10 of the original 12 subjects were still available to move on to a control condition (one male and one female were deceased by this stage). The control sessions were run after the test sessions so as to match the procedure of Engelmann and colleagues as closely as possible (Engelmann and colleagues did not run any control conditions). Each subject remained in his/her pre-assigned distributor group and underwent four *partner present* sessions. As with the test conditions, subjects had a maximum of one session per day, with 12 trials comprising a session (exceptions outlined in electronic supplementary material, table S3). The procedure in each control condition was identical to that of the equivalent partner present test condition; the only difference was that the partner monkey was now offered LV rather than HV food for each lever pull.

### 3.3. Coding

Three types of food refusals were coded (*refusal-to-participate*, *refusal-to-take* and *refusal-to-consume* a food item) and pooled during analysis to create a binary response variable (refusal = yes/no). In addition to food refusals, we coded pull-latency behaviour. These pull-latency data were used in an exploratory analysis. Fifty test trials and 43 control trials were excluded from analysis at the coding stage (documented in electronic supplementary material, tables S2 and S3). In total, 1066 test trials and 437 control trials were analysed.

E2 coded all videos and a second coder who was blind to the hypothesis of the study coded 20% of the videos. Observer agreement on *refusal* behaviour was excellent (Cohen's kappa (κ) = 0.974). Agreement on *pull-latency* behaviour was also excellent (Pearson correlation coefficient = 0.999). A paired *t*-test indicated that one coder systematically logged shorter latencies than the other (*p* < 0.001), however with a mean difference of 0.102 s, the difference between the two coders is negligible.

### 3.4. Statistical analysis

We carried out three analyses in R ([34], version 4.1.1). In analysis one, we tested whether distributor, partner presence and/or their interaction had an effect on monkeys' food refusal behaviour during test conditions. To this end we constructed Model 1A, a generalized linear mixed model (GLMM; [35]; output in tables 1 and 2). The response variable was food refusal (yes/no), and hence the model was fitted with binomial error structure and logit link function [36]. Model 1A consisted of a two-way interaction between distributor and partner presence, in addition to their fixed main effects and the fixed effects of trial number, session number (consecutive numbering across partner present and absent conditions), sex and order (whether the subject began with the partner present or the partner absent test condition). Although it is not explicitly called for by any of the three hypotheses, we included the interaction term (distributor × partner presence) so as to match the analytic approach of [31]. The prediction of the social disappointment hypothesis in Engelmann *et al*. [31, p. 2]) is about a main effect of distributor, not about an interaction. Hence, the main effect of distributor remains of theoretical interest, and we can only assume that the interaction between distributor and partner presence was included to take care of the possibility that refusal behaviour in the different distributor conditions may be differentially affected by partner presence. Subject ID and subject-session ID were included as random effects. The latter was nested within subject and accounted for potential session-to-session variation in refusal probabilities. The model included random slopes [37,38] of partner presence, trial number and session number within subject, and trial number within subject-session ID. We included estimates of the correlations between the random intercept and slopes in the model. For Model 1A, we used the full dataset; 1066 trials collected in 93 sessions. The data were from 12 subjects tested with two partners.

**Table 1.** Model 1A (full model): testing the effect of the interaction between distributor and partner presence as well as their main effects and the fixed effects of trial, session, sex and order on the probability to refuse LV food under conditions of inequality (table shows estimates, s.e., confidence intervals, likelihood ratio test (LRT), test results and minimum and maximum of model estimates obtained after dropping levels of random effects one at a time)*.*

term	estimate	s.e.	CI_Lower_	CI_Upper_	LRT	d.f.	*p*-value	min.	max.
intercept	−3.650	0.805	−5.342	−2.222			^a^	−4.460	−3.016
distributor (machine)	−1.503	0.831	−3.309	0.101			^a^	−2.485	−0.474
partner presence (yes)	−0.058	0.779	−1.521	1.531			^a^	−0.971	0.412
trial^b^	0.192	0.276	−0.389	0.738	0.450	1	0.502	0.022	0.484
session^c^	0.422	0.357	−0.246	1.273	1.396	1	0.237	0.248	0.627
sex (male)	1.253	0.613	0.053	2.423	3.332	1	0.068	0.863	2.032
order (partner)	2.409	0.587	1.175	3.985	11.825	1	0.001	2.066	3.264
distributor : partner presence	−2.133	1.255	−6.669	0.312	3.168	1	0.075	−3.067	−1.030

^a^Not indicated as of limited interpretability.

^b^z-transformed to a mean of 0 and a standard deviation of 1; mean and s.d. of the original variable were 6.506 and 3.448, respectively.

^c^z-transformed to a mean of 0 and a standard deviation of 1; mean and s.d. of the original variable were 4.412 and 2.275, respectively.

**Table 2.** Model 1A (reduced model lacking the interaction between partner presence and distributor): testing the effect of distributor, partner presence, trial, session, sex and order on the probability to refuse LV food under conditions of inequality (estimates, s.e., confidence intervals and test results).

term	estimate	s.e.	CI_Lower_	CI_Upper_	LRT	d.f.	*p*-value
intercept	−3.186	0.764	−4.967	−1.733			^a^
distributor (machine)	−2.571	0.672	−4.210	−1.334	9.066	1	0.003
partner presence (yes)	−0.795	0.637	−1.992	0.420	1.455	1	0.228
trial^b^	0.239	0.277	−0.321	0.757	0.689	1	0.407
session^c^	0.400	0.363	−0.312	1.104	1.207	1	0.272
sex (male)	1.246	0.635	0.087	2.610	3.074	1	0.080
order (partner)	2.350	0.608	0.954	4.083	10.434	1	0.001

^a^Not indicated as of limited interpretability.

^b^z-transformed to a mean of 0 and a standard deviation of 1; mean and s.d. of the original variable were 6.506 and 3.448, respectively.

^c^z-transformed to a mean of 0 and a standard deviation of 1; mean and s.d. of the original variable were 4.412 and 2.275, respectively.

To ascertain the effect of our factors of interest (distributor, partner presence and the interaction between distributor and partner presence), we used a likelihood ratio test to compare the full model outlined above with a null model lacking the interaction term and their main effects. Such a full–null model comparison aims to control for ‘cryptic multiple testing’ [39], which arises from the fact that we have three terms of interest in the model rather than just one. If the full–null model comparison reveals significance it means that among the terms present in the full and absent in the null model, there is at least one term which significantly contributes to the response. Such a test keeps the type one error rate at the nominal level of 0.05. The error rate would be inflated if one were to only inspect the significance of the individual effects [39].

Given that we used two partner monkeys in the course of the experiment, we fitted a second, identical model—Model 1B—using just the data obtained with partner identity 1, in order to check whether partner identity had an obvious effect on the outcome. For Model 1B, we used 597 test trials collected in 52 sessions. The data came from 12 subjects tested with one partner. For more detail and to see the output of Model 1B, see electronic supplementary material, tables S5 and S6.

In a second analysis, we considered the control condition data. As our control conditions were run subsequent to the test conditions (so as to emulate the Engelmann *et al*. [31] study design as closely as possible), we have taken care to consider the control conditions in a separate analysis and appreciate that we need to treat these findings with some caution. In this analysis, we tested whether the value of the partner's reward had an effect on monkeys' food refusal behaviour in the two distributor conditions. More specifically, we tested whether the subjects were more or less motivated to accept the LV reward in the human distributor as opposed to the machine distributor condition. To this end, we constructed another GLMM—Model 2 (output in electronic supplementary material, table S7). The response variable was food refusal (yes/no). The model comprised the fixed effects of distributor, trial number, session number and sex. Subject ID and subject-session ID were included as random effects and random slopes of trial number and session number within subject and trial number within subject-session ID were included. The same monkey was the partner in all control conditions and we therefore did not need to consider partner ID in the model. For Model 2, we used 437 control condition trials. The trials were collected in 40 sessions and the data came from 10 subjects with one partner. To ascertain the effect of our factor of interest (distributor), we used a likelihood ratio test to compare the full model outlined above with a null model that was identical to the full model in all respects except that it lacked this factor of interest (distributor).

In a third analysis, we explored the pull-latency data to establish whether the time it took a monkey to pull the lever differed as a function of distributor, partner presence or an interaction between distributor and partner presence. Here we fitted a survival analysis—Model 3 (documented in electronic supplementary material, §7.2, tables S8 and S9). The response variable was time in seconds, determined as when the subject pulled the lever or when the trial ended. Distributor and partner presence were included as an interaction term (in addition to their main effects), and trial number, session number, sex and order (whether the subject began with the partner present or the partner absent test condition) were included as fixed effects. Subject ID and subject-session ID were included as random effects. Random slopes of partner presence, trial number and session number within subject were included, in addition to the random slope of trial number within subject-session ID. Model 3 used the full dataset of 1066 trials; of these, 119 trials were *refusal-to-participate* (i.e. ‘timeout’) events. To ascertain the potential interactive effect of distributor and partner presence on pull-latency, we conducted a likelihood ratio test, comparing the full model with a null model lacking the interaction term and their main effects.

Where appropriate, we checked model stability and collinearity (inspected variance inflation factors; [40]). Confidence intervals, where reported, were derived using the *bootMer* function of the *lme4* package. More details on the statistical analyses can be found in the electronic supplementary material (§7).

Two notes on our decision-making process during analysis: in cases where an interaction of interest returned a trend-level effect (i.e. returned a *p*-value between 0.05 and 0.1), we could neither ignore nor unequivocally assume the possibility of an effect. In the face of such an event, we report the interaction term effect as our main finding but we take the extra step of fitting and reporting the output of the reduced (additive) model, i.e. we provide the reader with the complete information. We take a similar approach in the face of full–null model comparisons that just fail to reach significance; here we can again neither ignore nor unequivocally assume the possibility of a significant difference between the two models and for this reason we take the step of fitting and reporting the reduced (additive) model in addition to reporting the full model.

## 4. Results

### 4.1. Effect of distributor and partner presence

**Figure 2.**
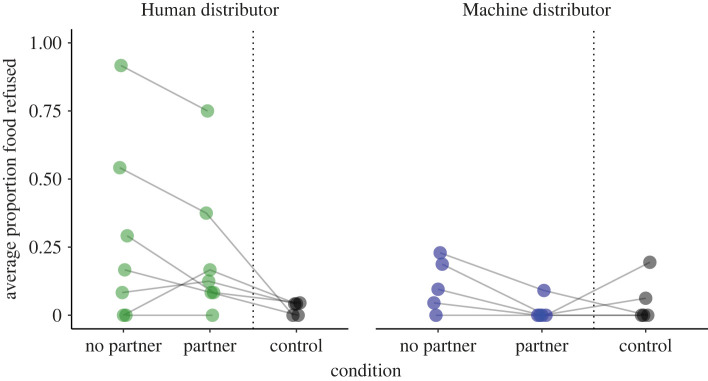
Data points show the median proportion of food refused per subject, according to distributor and condition (test conditions: *N*_Human_ = 7, *N*_Machine_ = 5, control condition: *N*_Human_ = 5, *N*_Machine_ = 5). Test conditions and the respective control conditions are separated by a dotted vertical line to emphasize that the control conditions were run after the test conditions and are assessed in a separate analysis.

Monkeys in the human group rejected on average (median) 16.7% of the food within a session when a partner was present and 25% of the food when a partner was absent (figure 2). Monkeys in the machine group rejected on average no food within a session when a partner was present and 4.2% of the food when the partner was absent. To establish whether an interaction between *distributor* and *partner* presence affected refusal behaviour we constructed a GLMM, Model 1A. Comparison of the full with the null model revealed a significant difference between the two models (likelihood ratio test: $\chi_{3}^{2}=14.226$, *p* = 0.003). Inspection of the full model output (table 1) revealed that there was a trend for an interactive effect of distributor and partner presence on food refusal behaviour (*p* = 0.075). This finding means any interpretation of the main effects of distributor and partner presence needs to be done with caution, as the main effects are potentially only meaningfully interpretable as part of this interaction. The output of a reduced (additive) model (table 2) revealed a significant effect of distributor, with monkeys in the human distributor condition more likely to refuse food compared with those in the machine distributor condition.

In a side finding, the full model revealed a trend for sex (*p* = 0.068). Male monkeys showed higher refusal rates (human distributor: 41.7%, machine distributor: 0%) than female monkeys (human distributor: 8.3%, machine distributor: 0%). Furthermore, monkeys who experienced the presentation order ‘present-present-absent-absent-present-present-absent-absent’ refused on average in 16.7% of trials, while monkeys with the opposite presentation order refused in 0% of trials (effect of order: *p* = 0.001).

### 4.2. Control condition

To test whether there was an effect of distributor when the partner received the same LV food as the subject (figure 2, control conditions), we fitted Model 2. A full–null model comparison revealed no significant difference between the two models (likelihood ratio test: $\chi_{3}^{2}=<0.001$, *p* = 0.984).

### 4.3. Pull-latency

**Figure 3.**
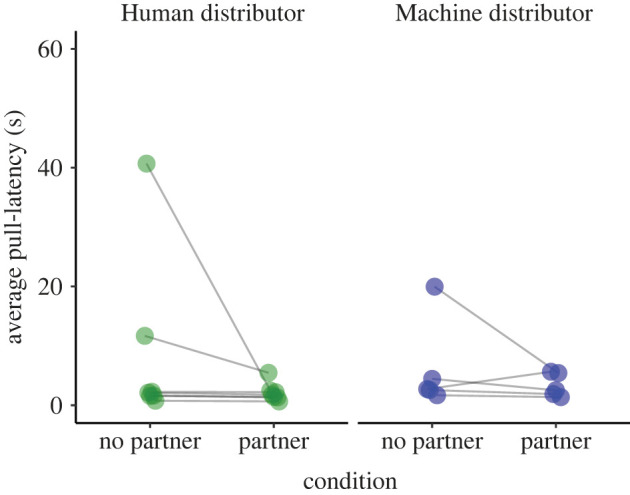
Average (median) pull-latency (time in seconds) between the table light coming on and the subject pulling the lever; each monkey was given 120 s to pull the lever. If the monkey refused to participate within this time, the experimenter turned the light off and blocked the lever. In total, there were 119 *refusal-to-participate* events. Figure 3 therefore displays 947 of 1066 pull-latencies. Data points show the median pull-latency for each subject in their respective test conditions (green data points: *N*_Human_ = 7, blue data points: *N*_Machine_ = 5).

Monkeys were faster in pulling the lever in the partner present as opposed to the partner absent conditions (figure 3). In the partner present test conditions, the median pull-latency was 2.26 s (human distributor median: 2.04 s; machine distributor median: 2.52 s), while in the partner absent test conditions, the median pull-latency was 3.4 s (human distributor median: 2.76 s; machine distributor median: 4.22 s). To explore whether subjects' pull-latencies varied according to test condition, we fitted a survival analysis—Model 3. A full–null model comparison revealed a trend-level difference between the two models (likelihood ratio test: $\chi_{3}^{2}=6.871$, *p* = 0.076). Given the marginally non-significant difference between the full and the null models, we proceeded to inspect the contribution of the highest order predictor term in the full model.

The interaction between distributor and partner presence was not significant (*p* = 0.392; full model output in electronic supplementary material, table S8) and we therefore ran a reduced (additive) model (output in electronic supplementary material, table S9). The reduced model revealed a significant effect of partner presence on pull-latency; monkeys were quicker to pull the lever when the partner was present in the neighbouring test compartment as compared with when the neighbouring compartment was empty.

As a side finding, the reduced model revealed a significant effect of trial (*p* = 0.017); as trial number increased, pull-latencies became longer. Furthermore, the reduced model revealed a significant effect of sex (*p* = 0.020); males took longer to pull the lever compared with females. Finally the output revealed a trend-level effect of session (*p* = 0.084); as session number increased, pull-latencies became longer.

## 5. Discussion

The present study investigated whether the social disappointment hypothesis could explain the food refusal behaviour of a catarrhine species in an inequity aversion paradigm. The task for our subjects was to pull a lever to obtain food rewards. They did so either with a conspecific co-worker in the adjacent test compartment, or when alone. Furthermore, rewards were distributed either by a human experimenter, who would place a food item onto a sliding platform that moved within the subjects’ reach, or distribution was automated and no human experimenter was present. While the partner (or the empty cage) was provided with HV food rewards, subjects always received LV food rewards. We were interested in subjects' refusal to take the food or pull the lever as a function of experimental context (i.e. distributor type and partner presence). We found that monkeys in the human distributor condition showed higher refusal rates than monkeys in the machine distributor condition, although this main effect of distributor has to be interpreted alongside our finding of a (weak) interactive effect of distributor and partner presence on food refusal. Subjects were slightly faster in pulling the lever when the conspecific partner was present, as reflected by a marginal difference between the full and the null models. In our control conditions, when both individuals received LV food rewards, we found very low refusal rates in both distributor conditions. In what follows, we consider how well our data fit each account in turn (inequity aversion, food-centred and social disappointment) before making a suggestion as to what our results may imply.

First, the data of our experimental conditions do not provide support for the inequity aversion hypothesis, which predicts that monkeys in both distributor groups should have fixated upon their partner's pay-off and refused food more often in the presence of a partner. We report a trend-level interaction between distributor and partner presence and from looking at the raw data, it is clear that the effect of partner presence is in the opposite direction to the pattern expected by the inequity aversion account. In the control conditions, we observed very low refusal rates irrespective of distributor type. On the one hand, this fits with predictions of the inequity aversion hypothesis—both partners are treated alike, hence there is no reason to protest by refusing the offered food. On the other hand, the low refusal rate in the human distributor control condition may be due to a slight difference between the human test and control conditions: in the test conditions, the experimenter reached to take food from the partner's HV cylinder. Seeing this could have triggered the subject's expectation of receiving HV food. By contrast, in the control condition, the experimenter only ever reached for the LV food, hence no expectation of HV food was triggered or monkeys did not consider HV food to be a possible option for the experimenter to reach for and provide.

Second, our data do not support either of the food-centred hypotheses. The food expectation hypothesis predicts that subjects should refuse LV food in conditions where HV food is visible [24]. In our study, subjects saw both HV and LV food in each condition, including the control conditions. If they only behaved according to the food expectation hypothesis, then the refusal rates should have been equally high in all conditions; this was not the case. The frustration hypothesis predicts that subjects should refuse LV food after having received HV food. In steps 3 and 4 of the familiarization procedure, subjects had experienced that ‘work’ (pulling the lever) could lead to a HV reward. If this experience was enough to induce a ‘contrast effect’ [41], then subjects' frustration should have affected refusal rates similarly in all four experimental conditions; as stated, this was not the case. Anecdotally, observation of the partner's behaviour suggests that a contrast effect might appear after a higher number of trials: our partner, who was used to receiving HV food during the test trials, was extremely difficult to motivate to participate in the control trials, where both monkeys received LV food. Future studies might look into the induction of the contrast effect in more detail.

Third, results from our experimental conditions are compatible with the social disappointment hypothesis, which predicts that monkeys in the human distributor condition expect to be provided with HV food and consequently will refuse LV food at higher rates when a human distributes the food compared with a machine. Our main finding was of a trend-level interaction between distributor and partner presence. The social disappointment hypothesis implies that monkeys refuse food because they hold certain expectations toward the human based on the past experience of having received food, while monkeys in the machine group have no such expectations with regard to an inanimate distributor. In contrast with predictions of the social disappointment hypothesis, however, there was no evidence for an effect of distributor in the control condition, where refusal rates were extremely low in both conditions.

The predictions we laid out for the different explanatory accounts reflect the pure forms of these accounts without additional amendments. Our finding of a trend-level interactive effect of distributor and partner presence, as well as the distribution of the raw data and the strong effect of distributor in the reduced model, support the social disappointment hypothesis. The extremely low refusal rates observed in both distributor control conditions do not match the social disappointment account. The low refusal rates in the control conditions fit better with either a lower level explanation or the inequity aversion account—in these conditions both partners are treated the same, so there is no unfairness against which to protest. Taken together, what we think the results show is that more than one factor is responsible for the monkeys' behaviour in our experiment.

We suggest that underlying food competition and/or social facilitation effects may be modulating the subjects’ behaviour via decreased reaction times when a partner was present (see also electronic supplementary material, model output, tables S8 and S9). Here, however, in light of the marginally non-significant test statistic returned following the full–null model comparison (likelihood ratio test), further investigation is needed before we can conclude this with any certainty. Interestingly, Engelmann *et al*. [31] reported a trend whereby chimpanzees in the human distributor condition refused less often when a partner was present compared with when alone in the test cage. The authors surmised that this effect was due to competition over food, i.e. that watching a conspecific consume food may influence a subject's resolve or inhibitory control and heighten competitive arousal [31,42]. Ample evidence demonstrates that non-human primates increase food intake when in social as opposed to alone conditions [43,44], and the sight and sound of a monkey consuming food nearby is thought to activate a similar motor programme in an observing monkey [45]. Hence, in addition to disappointment in the experimenter, other aspects such as attention to social partners as well as lower level social faciliation/competition, affect non-human primates' responses in these types of inequity aversion scenarios.

In the current study, the human distributor was someone the monkeys have interacted with previously and with whom they have a positive relationship. In follow-up studies, it would be interesting to manipulate the experimenter–subject relationship to see whether subjects’ disappointment levels fluctuate systematically. For example, they might hold stronger expectations about being treated well towards familiar researchers who have frequently provided HV food in the past, than towards novel researchers, and might consequently refuse LV food more often when it is provided by the familiar experimenter. A number of studies have investigated non-human primate sensitivity to reputation [46,47] and to the reliability of the humans they interact with (e.g. [48]) and have shown that the subjects can take social information about different people into account: they prefer nice over mean and reliable over unreliable experimenters. It would be interesting to contrast how animals responded to the familiar human, with how they would respond to a neutral human or a human they view negatively. In addition, animals with more test experience should have stronger expectations around being treated with concern compared with more naive monkeys. Studies such as these would help to illuminate how the monkeys form expectations.

One limitation to the present study was the fact that the human–monkey interaction was rather artificial. So as to be systematic between the two distributor conditions, we held many aspects of the food delivery constant, i.e. the food always arrived via the platform that travelled on the conveyor belt to within reach of the monkey. Holding the method of delivery constant between the two distributor conditions had its advantages, e.g. we were able to standardize the speed of the food delivery. However, it is possible that creating human–monkey interactions that are more ‘natural’ for this study group, e.g. having the human offer the reward directly to the monkey, might mediate the levels of protest behaviour in the human distributor condition. Future studies might manipulate aspects of the human–monkey interaction in an effort to establish whether different action sequences mediate subjects' protest behaviour.

Another interesting aspect for future research might be to explore the scope of applicability of the social disappointment account. Theoretically, the account, as presented in Engelmann *et al*. [31] only has a very narrow application, namely experimental ‘inequity aversion paradigms'. The authors make no suggestions regarding a potential broader applicability; hence, while providing an interesting alternative explanation of animal's behaviour in a specific situation, the broader relevance of the account remains unclear. Empirically, interesting open questions pertain to how the predictions of the account play out for different captive groups. For example, captive groups differ in the amount of interaction they have with humans and the feeding regime they usually experience. The chimpanzees of Engelmann *et al*. as well as the long-tailed macaques of the current study are both routinely fed directly by human caregivers and have a history of participating in interactive behavioural experiments with researchers. But other captive populations might experience fewer regular interactions with humans and might even regularly experience receiving food from automated food dispensers. Testing a group like this might be another interesting test case for the social disappointment account. Engelmann *et al*. explain that social disappointment stems from having established a certain dyadic relationship with the researcher. This implies that, if no such relationship is established, no social disappointment should arise that would cause the respective frustration behaviours. Other test cases involve the reactions towards humans who are known to be unreliable food providers or known in unpleasant roles (e.g. the local vet).

With our limited sample size, we could not systematically assess effects of individual variables such as, for example, nutritional status, personality traits, rank or individual decision-making profiles (i.e. risk preference tendencies or ability to delay gratification). These and many other aspects could potentially modulate whether an individual acts on their perceived social disappointment or disadvantageous reward treatment. Future research will have to explore the effects of such individual subject variance in relation to group effects.

In summary, the long-tailed macaques' behaviour in the current study can be best explained by a combination of social disappointment in the human experimenter and a certain degree of food competition and/or social facilitation. The response patterns resemble previous findings in chimpanzees and thus extend the social disappointment account as one important facet in refusal behaviour beyond the ape lineage to a catarrhine species.

## Data Availability

Supporting material is supplied in electronic form [49]. The datasets and R code which accompany this paper have been made available on Dryad Digital Repository: https://doi.org/10.5061/dryad.3n5tb2rnb [50].
